# Editorial: Innate immune evasion strategies during microbial infection

**DOI:** 10.3389/fcimb.2023.1332253

**Published:** 2023-11-10

**Authors:** Selvakumar Subbian

**Affiliations:** Public Health Research Institute Center at New Jersey Medical School, Rutgers University, Newark, NJ, United States

**Keywords:** innate immunity, host response, microbial infection, pathogens, vaccines, host-pathogen interactions

Innate immunity is the primary host mechanism of protection against infectious diseases in humans and animals. This broad and non-specific response fends off the initial infection and facilitates the establishment of pathogen/antigen-specific adaptive immunity. Thus, innate immunity is crucial in determining the outcome of host-pathogen interactions. Vaccination with non-infectious molecular components of pathogens boosts the host immunity to respond effectively against infectious pathogens. However, pathogens of humans and animals have evolved with mechanisms to counteract the hostility and persist within the infected host. Ultimately, the combined innate, adaptive, and vaccine-induced immunity impacts the host-pathogen interactions and subsequent course of infection. In this Research Topic, five research articles and a mini review are presented on various host-pathogen factors and vaccine components that affect bacterial and viral pathogenesis in humans and animals.

*Aggregatibacter actinomycetemcomitans* (Aa) is an etiological agent of localized aggressive periodontitis (LAP) in humans (Gholizadeh et al., 2017). The cytolethal distending toxin (Cdt) is among the various virulence factors produced by Aa to cause disease in the oral cavity (Boesze-Battaglia et al., 2016). The Cdt of Aa depletes the pool of phosphatidylinositol triphosphate (PIP3) through its phosphatase activity, thus blocking the activation of inflammasome and subsequent cytokine production by the host cells (Shenker et al., 2016). Since phosphoinositides (PI) are crucial in phagosome-lysosome fusion and pathogen clearance, perturbing the PI pool may confer a host-evasion mechanism. In this Research Topic, Kim et al. reports that Aa employs Cdt to manipulate the PI pool of phagosomes, which subsequently impacts the association of phagosomes with Rab5, a key endocytic pathway molecule. Specifically, Kim et al. demonstrated that THP-1-derived macrophages displayed impaired phagosome maturation and phagolysosome formation upon exposure to Cdt from Aa. This effect further compromises the phagocytosis and antimicrobial effects of the macrophages, resulting in elevated growth of Aa in infected macrophages. Further mechanistic studies from similar Cdt in other pathogenic bacterial species should inform the precise role of this virulence factor in causing disease and how it is regulated between health and disease.

Salmonellosis, caused by non-typhoidal Salmonella serovars such as *Salmonella enterica. Ser. Enteritidis*, is a common bacterial disease in humans worldwide (Mkangara, 2023). Importantly, no effective vaccine is currently available to protect against *S. Enteritidis* infection (Ali et al., 2023). In this Research Topic, Jiang et al, developed a novel outer membrane vesicle (OMV)-based vaccine for *S. Enteritidis* infection and tested its protective efficacy in a murine model. Specifically, the authors have created mutants of *S. Enteritidis* defective for *tolR* (Δ*tolR*), a gene that codes for the inner membrane protein, and *rfaQ* (Δ *rfaQ*) that codes for a transferase involved in keeping the stability of the outer membrane and evaluated their OMV production, compared to the wild type (WT) bacteria. It should be noted that previous studies have shown that a mutant *S. Enteritidis rfaQ* was defective for virulence in model systems, while a *tolR* mutant displayed elevated expression of OMVs in other Salmonella species (Yethon et al., 1998; Nevermann et al., 2019). Consistent with these reports, more OMVs were extracted from the Δ*tolR* strain, which was higher than that obtained from Δ *rfaQ*, but comparable to the yield from WT. Similarly, when used as a vaccine, the OMV of both WT and Δ*tolR* strains provided better protection than the OMV Δ*rfaQ* strain in mice orally challenged with *S. Enteritidis*. However, bacterial clearance from infected tissues was more effective in infected mice that were previously vaccinated with OMV from WT or Δ *rfaQ* strains, although immunization with OMV from each strain produced similar levels of humoral immunity. Thus, this study highlights that tweaking the OMV production machinery may be an effective strategy for vaccine development against *S. Enteritidis* infections.

Two of the articles in this Research Topic, by Wu et al. and  Zhao et al. respectively, pertain to infectious diseases of pigs. Wu et al constructed 32 monoclonal antibodies (mAbs) against the recombinant outer membrane protein P2 (rOmpP2) of various *Haemophilus parasuis* genotypes, which causes Glasser’s disease in pigs (Oliveira and Pijoan, 2004). These OmpP2 are immunodominant antigens and elicit a high inflammatory and antibody response in the host, thus facilitating effective host control of the infection (Wu et al., 2017). However, the mechanistic role of OmpP2 on host pathogenesis is not well understood. In the current study, 32 rOmpP2 mAbs were tested for their reactivity to a panel of genotype-common and genotype-specific OmpP2 peptides. From this, several broad and genotype-specific B cell epitopes were selected and tested using positive sera from mice and pigs and in the macrophage model to delineate the functional correlation of the OmpP2 with corresponding immunogenicity. Results from this study suggest the possibility of discriminating the virulence of *H. parasuis* based on the OmpP2 genotype, which is consistent with prior studies (Yu et al., 2014). Since the OmpP2 exerts a strong proinflammatory and antibody response, they may be considered for candidate subunit vaccines to use against *H. parasuis* infection. In the second article, Zhao et al. developed three DNA vaccines against the porcine reproductive and respiratory syndrome virus (PRRSV), the causative agent of porcine high fever disease (Lunney et al., 2010). The selected DNA vaccines, targeting PRRSV GP3 and GP5, when formulated with a saphonin extract combined with a water-in-oil-in-water adjuvant showed a high level of immunogenicity and protective efficacy in a pig model of PRRSV infection, although the virus was still detectable in the tissues of vaccinated and challenged pigs, suggesting the need for further characterization/optimization studies.

The differential immune niche between systemic and the gut during bacterial infection was evaluated in a *Manduca sexta* model by von Bredow et al. Findings of this study suggest that systemic *E. coli* stimulation is unlikely to establish immunity in the gut, while midgut infection with *B. thuringiensis* activates immune response in systemic organs.

Finally, the mini review by de Almeida et al. summarizes the role of lipid droplets (LD) during mycobacterial infections. Since pathogenic mycobacteria can exploit the host cell lipid metabolism for survival and infection, understanding the immune-modulatory role of LDs on immune cells through various mechanisms is crucial for identifying potential targets to control mycobacterial infections.

In summary, this Research Topic includes articles on the molecular immunological determinants of host-pathogen interactions using various model systems of bacterial and viral infections. The concepts and findings presented in these articles would provide a platform for devising better and improved interventions for infectious diseases.

## Author contributions

SS: Writing – original draft, Writing – review & editing.
